# Prevalence and Severity of Sensorineural Hearing Loss in Diabetic and Hypertensive Patients: A Comparative Cross-Sectional Study

**DOI:** 10.7759/cureus.62573

**Published:** 2024-06-17

**Authors:** Mahmood Mohiuddin Mohammed, Abdul Majid Shaik, Zoya Riyaz Syeda, Rohit Khare, Suswara Bukka, Aarfa Devani, Lakshmi Tejaswi Sakhamuri, Ruqiya Bibi, Muhammad Subhan, Saifullah Syed

**Affiliations:** 1 Internal Medicine, Kamineni Academy of Medical Sciences and Research Centre, Hyderabad, IND; 2 General Medicine, Kakatiya Medical College, Warangal, IND; 3 General Medicine, Faculty of Medical Sciences, Khaja Bandanawaz University, Gulbarga, IND; 4 Family Medicine, Rajiv Gandhi Institute of Medical Sciences, Adilabad, Adilabad, IND; 5 Internal Medicine, Malla Reddy Institute of Medical Sciences, Hyderabad, IND; 6 Internal Medicine, Kamineni Academy of Medical Sciences and Research Center, Hyderabad, IND; 7 Internal Medicine, Jinnah Hospital, Allama Iqbal Medical College, Lahore, PAK; 8 Internal Medicine, Royal College of Surgeons in Ireland, Dublin, IRL

**Keywords:** hypertension, degree of hearing loss, moderate hearing loss, hearing function, pure tone audiometry, diabetes, sensorineural hearing loss

## Abstract

Background

The ability to perceive sound is crucial for effective communication and environmental awareness. This study aimed to assess sensorineural hearing loss (SNHL) in patients with both diabetes mellitus (DM) and hypertension (HTN).

Materials and methods

A total of 120 participants of both genders were divided into three groups: Group I consisted of diabetic patients (n=40, 22 males and 18 females), Group II included hypertensive individuals (n=40, 16 males and 24 females), and Group III served as controls (n=40, 15 males and 25 females). General ear examinations, including otoscopy, were conducted. Hearing function and the degree, pattern, and configuration of hearing loss were assessed using general ear examinations and pure tone audiometry.

Results

Normal hearing was observed in 25% of Group I, 26% of Group II, and 85% of Group III. Mild hearing loss was evident in 75% of Group I, 50% of Group II, and 15% of Group III, while moderate hearing loss was observed in 24% of Group II. These differences were statistically significant (P < 0.05).

Conclusion

Diabetic and hypertensive individuals demonstrated a higher hearing loss prevalence than healthy controls.

## Introduction

Auditory perception, or hearing, represents a fundamental sensory function crucial for human interaction and environmental awareness [1]. The intricate process of hearing involves detecting and interpreting sound vibrations transmitted through the ear, facilitating communication and situational awareness [2]. Hearing loss, characterized by a diminished ability to perceive sound, poses significant challenges to individuals' daily lives, affecting communication, social interaction, and overall quality of life [1,2]. Acquired hearing loss, distinguished from a congenital impairment, arises from various environmental factors, including exposure to hazardous substances such as chemicals, medications with ototoxic properties, and prolonged exposure to excessive noise [3]. This hearing impairment often damages delicate auditory structures within the inner ear, including hair cells, supporting cells, and neural pathways, highlighting the complex interplay between environmental factors and auditory function [3]. Recent epidemiological studies have underscored a concerning trend linking certain medical conditions, such as diabetes mellitus (DM) and hypertension (HTN), to an elevated risk of sensorineural hearing loss (SNHL) [4-8]. Notably, individuals with DM are more susceptible to auditory impairment, particularly affecting higher frequency tones, although the specific mechanisms accountable for this correlation are not yet entirely understood [5]. However, limited clinical evidence suggests a potential link between DM and hearing loss, necessitating further investigation to elucidate causal relationships and identify potential therapeutic interventions [6].

Moreover, the impact of comorbid conditions, aging-related changes, and environmental exposures on hearing function in diabetic populations warrants careful consideration to accurately assess the scope of this issue and inform targeted management strategies [6,7]. In addition to DM, HTN has emerged as another significant risk factor for SNHL, prompting interest in exploring the interplay between cardiovascular health and auditory function [8]. HTN-related vascular changes, microvascular damage, and altered blood flow dynamics may contribute to cochlear pathology, resulting in SNHL [8]. However, previous studies' conflicting evidence and methodological limitations underscore the need for comprehensive investigations incorporating robust study designs and meticulous control of confounding variables to elucidate the precise mechanisms linking HTN to auditory dysfunction [9-11]. Furthermore, the potential synergistic effects of DM and HTN on hearing loss merit attention, as individuals with both conditions may face compounded risks and unique challenges in managing their auditory health [12].

This study assesses the prevalence and severity of SNHL in diabetic and hypertensive patients compared to healthy controls. Objectives include measuring pure tone audiometry (PTA) thresholds, comparing hearing thresholds among the three groups, analyzing the correlation between the duration of DM or HTN and the degree of hearing loss, and exploring mechanisms and risk factors linking DM, HTN, and SNHL.

## Materials and methods

This was a cross-sectional observational study to assess sensorineural hearing loss in patients with DM and HTN. The Institutional Review Board of the Kamineni Academy of Medical Sciences and Research Centre reviewed and approved the study (approval number: KAMSRC/IRB/2023/10-324), ensuring the protection of ethical procedures and participant rights. The research followed the ethical principles outlined in the Declaration of Helsinki and was approved by the hospital's review board. Before participating, all people gave informed consent before enrollment.

Study participants

The study population consisted of 120 subjects of both genders, aged between 40 and 60, who attended the hospital's outpatient clinic between October 2023 and March 2024. To differentiate from presbycusis, which is age-related hearing loss, this study's subjects were selected based on specific medical conditions (DM and HTN) and excluded those with factors that typically contribute to presbycusis, such as a history of loud noise exposure or ototoxic drug use. This distinction allows for a clearer association between the medical conditions studied and hearing loss, separate from the natural aging process.

The participants were separated into three groups based on their medical background and laboratory tests: Group I had 40 patients with DM (22 males and 18 females), Group II had 40 patients with HTN (16 males and 24 females), and Group III had 40 healthy controls without DM or HTN (15 males and 25 females). Participants, evenly distributed within the 40-60 age range, had a near-equal gender distribution and diverse socioeconomic backgrounds. The inclusion criteria for Group I was a diagnosis of DM according to the American Diabetic Association criteria [13]. A fasting sugar level above 126 mg/dL or glycated hemoglobin (HbA1c) level of more than 6.5% and treatment with oral hypoglycemic agents or insulin. The inclusion criteria for Group II were: diagnosis of HTN per the criteria outlined by the American College of Cardiology/American Heart Association (ACC/AHA), including systolic blood pressure surpassing 130 mmHg or diastolic blood pressure exceeding 80 mmHg, along with treatment involving antihypertensive medication. The inclusion criteria for Group III were the absence of DM or HTN, normal blood glucose and blood pressure levels, and no history of chronic diseases or medications that could affect hearing function.

The duration and severity of DM and HTN were carefully assessed and categorized based on established criteria. The duration of DM or HTN was determined from patient self-reports and medical records, documenting the number of years since the initial diagnosis. The severity of DM was categorized according to HbA1c levels: Stage 1 (6.5-7.5%), Stage 2 (7.6-9.0%), and Stage 3 (above 9.0%). The severity of HTN was categorized based on the ACC/AHA guidelines: Stage 1 (systolic blood pressure 130-139 mmHg or diastolic blood pressure 80-89 mmHg) and Stage 2 (systolic blood pressure at least 140 mmHg or diastolic blood pressure at least 90 mmHg).

The exclusion criteria for all groups were: history of ear diseases, ear surgery, ear trauma, ear infections, ototoxic drug use, exposure to loud noise, family history of hearing loss, smoking, alcohol consumption, and other conditions that could interfere with hearing function.

Data collection

Data such as name, age, gender, height, weight, body mass index, medical history, medication use, and lifestyle habits were recorded for each subject using a structured questionnaire. Blood samples were obtained from each individual after an overnight fast and analyzed for blood glucose, HbA1c, serum creatinine, blood urea nitrogen, and lipid profile using standard laboratory methods. Blood pressure was measured with a proper cuff and mercury gauge manometer after 10 minutes of rest in a sitting position. The mean of three readings was recorded as the blood pressure value.

A general ear examination uses otoscopy to rule out external or middle ear pathology. Hearing function, degree, form, and configuration of hearing loss were determined through a general ear examination and pure-tone average (PTA). A calibrated MA 25 audiometer (Maico Diagnostics USA, Eden Prairie, Minnesota, United States) performed PTA in a soundproof booth. Air conduction thresholds were measured at frequencies ranging from 250 Hz to 8000 Hz, and thresholds for bone conduction were evaluated at 500, 1000, 2000, and 4000 Hz frequencies. Participants received instructions to respond whenever they heard a tone, regardless of its intensity. The environment was controlled for ambient noise, with testing conducted in a soundproof booth to prevent external sound interference. PTA was determined by averaging the air conduction thresholds across 500 Hz to 4000 Hz.

Definitions

SNHL was defined as a PTA of more than 25 dB in either ear, with a normal or near-normal bone conduction threshold. The severity of hearing impairment was categorized as follows: mild (26-40 dB), moderate (41-60 dB), severe (61-80 dB)), or profound (>80 dB). Hearing loss was classified as symmetrical or asymmetrical, and the configuration of hearing loss was classified as flat, sloping, rising, or notched.

Statistical analysis

The study results were compiled and subjected to statistical analysis using IBM SPSS Statistics for Windows, Version 22.0 (Released 2013; IBM Corp., Armonk, New York, United States). Descriptive statistics summarized the participants' demographic characteristics and clinical and audiometric data. The Kolmogorov-Smirnov test was used to know the normality of the data distribution. The groups' differences were analyzed using the chi-square test for categorical variables. The statistical association between the variables was examined using the Pearson and Spearman Correlation coefficient for continuous variables with normal and non-normal distribution. A p-value of less than 0.05 was considered statistically significant.

## Results

**Figure 1:**
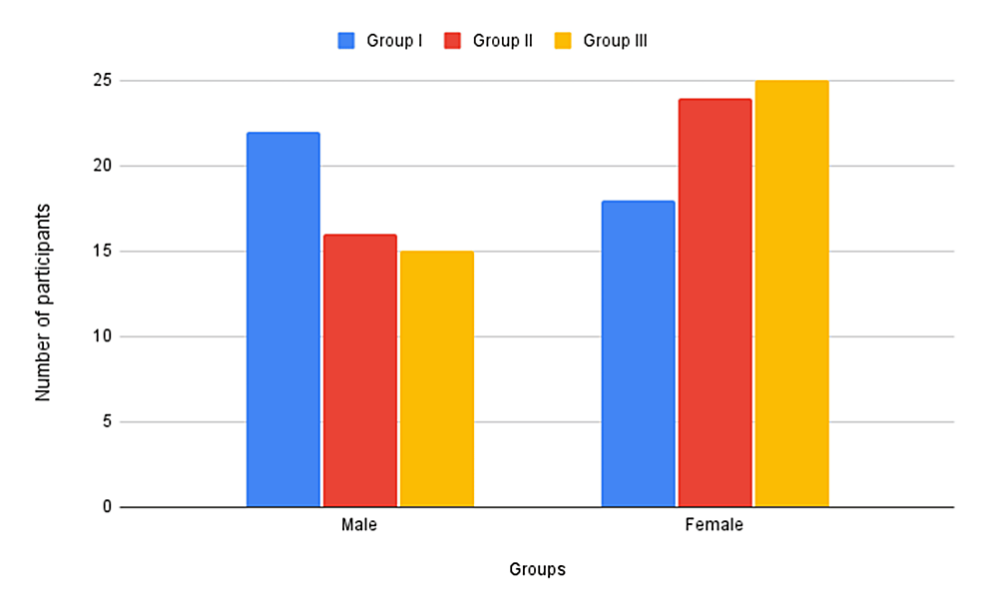
Distribution of subjects

The study included 120 participants, with an equal distribution of 40 individuals in each group (Figure 1). Group I had 22 males and 18 females, Group II had 16 males and 24 females), and Group III had 15 males and 25 females.

The mean age of the participants was 50 years, with a standard deviation (SD) of 5.7 years. There was no significant difference in age distribution among the three groups (p = 0.87). The participant's BMI reflects a population from normal weight to pre-obese categories. Table 1 depicts the demographic and clinical characteristics of these groups.

**Table 1:** Mean age and BMI of the groups Statistical analyses were performed using ANOVA to compare mean BMI among the three groups. The resulting F-ratio was 0.0445, significantly lower than the critical value (F-critical) of 19.00 at the 0.05 significance level. The p-value was 0.9574, indicating no statistically significant difference in mean BMI among the groups.

Groups	Mean Age (years) ± SD	Mean BMI (kg/m²)
I (Diabetes Mellitus)	50 ± 5.9	26.4
II (Hypertension)	50 ± 5.5	24.6
III (Controls)	50 ± 5.6	23.5

**Figure 2:**
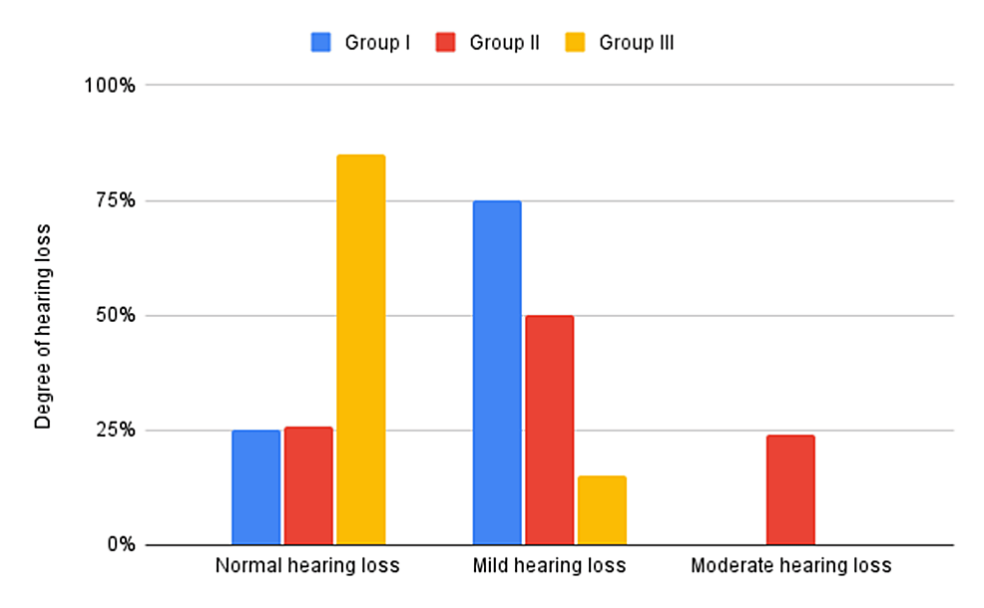
Assessment of sensorineural hearing loss in all groups

SNHL was observed in varying degrees across the groups (Figure 2). Normal hearing loss was observed in 25% of Group I, 26% in Group II, and 85% of Group III. Group I exhibited a higher prevalence of mild SNHL (75%) compared to Group II and Group III (controls), which showed 50% and 15%, respectively. Moderate SNHL was exclusively observed in Group II (24%).

Table 2 shows that the prevalence of SNHL varied significantly among the groups, as indicated by the chi-square test (χ² = 15.67, p < 0.05).

**Table 2:** Prevalence of sensorineural hearing loss Statistical significance was assessed using the Chi-square test. A p-value < 0.05 was considered statistically significant. SNHL: sensorineural hearing loss

Group	Mild SNHL (%)	Moderate SNHL (%)	p-value
I (Diabetes Mellitus)	75	0	0.02
II (Hypertension)	50	24	0.04
III (Controls)	15	0	0.01

PTA revealed that the average air conduction thresholds were significantly higher in Group I (35 dB) and Group II (30 dB) compared to Group III (20 dB). The most common audiometric configuration was a sloping type in Group I and a flat type in Group II. There was no significant asymmetry in hearing loss among the participants. Audiometric findings and asymmetry analysis across study groups are presented in Table 3.

**Table 3:** Audiometric findings and asymmetry analysis across study groups Statistical analyses were performed to assess asymmetry in audiometric configurations among the groups using the Chi-square test for independence. A p-value < 0.05 was considered statistically significant. The asymmetry p-values indicate no significant asymmetry in audiometric configurations among the groups, with all p-values (0.45, 0.30, 0.78) being greater than 0.05.

Group	Average Air Conduction Threshold (dB)	Most Common Audiometric Configuration	Asymmetry (p-value)
I (Diabetes Mellitus)	35	Sloping	0.45
II (Hypertension)	30	Flat	0.30
III (Controls)	20	None	0.78

**Figure 3:**
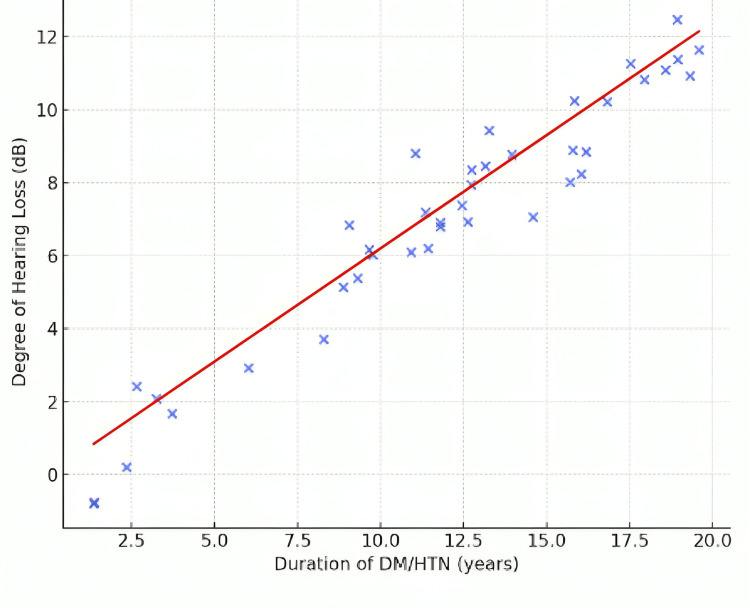
Correlation between duration of DM/HTN and degree of hearing loss DM: diabetes mellitus; HTN: hypertension

Figure 3 shows a positive correlation between the duration of DM and HTN and the degree of hearing loss (Pearson correlation coefficient = 0.62, p < 0.01).

**Figure 4:**
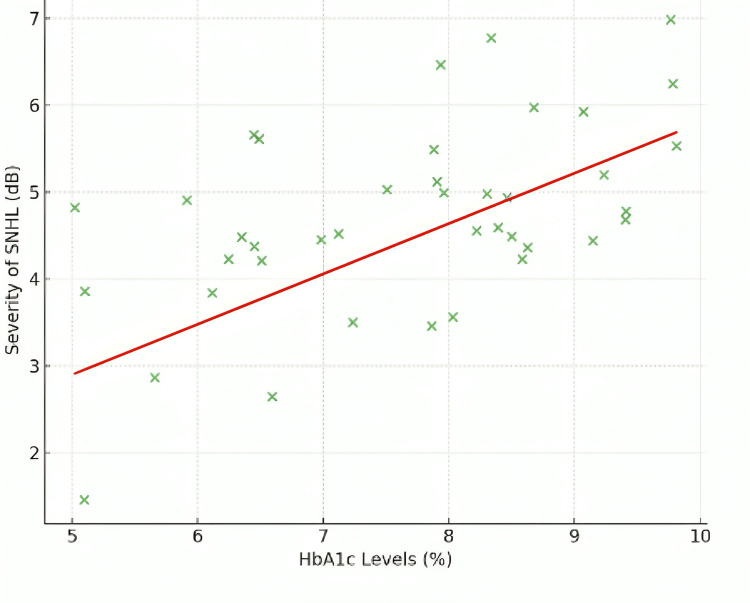
Correlation between HbA1c levels and severity of SNHL SNHL: sensorineural hearing loss; HbA1c: glycated hemoglobin

Additionally, a significant correlation was observed between higher HbA1c levels and the severity of SNHL (Spearman correlation coefficient = 0.58, p < 0.01), as depicted in Figure 4.

## Discussion

Hearing loss due to degenerative aging processes is known as presbycusis [7]. It is currently the most frequent sensory impairment observed in older people, with a prevalence ranging from 25% in the age group of 4-70 years, 50% in the elderly aged up to 85 years, and greater than 80% in people over 85 years of age [8]. Presbycusis can lead to diminished speech perception, psychological changes (such as depression), social isolation, problems related to alertness and defense (ability to hear automotive horns, telephone rings, and alarms), as well as cognitive functions [7,8]. All these factors harm older people's quality of life [9-14].

Systemic arterial hypertension (SAH) is a complex condition involving elevated blood pressure alongside metabolic and hormonal changes and trophic alterations phenomena (cardiac and vascular hypertrophy [10]. Dosemane et al., in their study in South India, found that a large proportion of diabetic patients had SNHL, with 90.2% having bilateral SNHL and 39% reporting ear complaints [11]. However, they found no significant association between hearing loss severity and diabetes-related factors such as glycemic control, duration of DM, or gender. Our cross-sectional study also focused on the association between DM and SNHL. However, it also included hypertensive patients and found a higher prevalence of hearing loss in both diabetic (75% mild, 24% moderate) and hypertensive (50% mild, 24% moderate) patients compared to healthy controls (85% normal hearing), with these differences being statistically significant. While both studies confirm a higher prevalence of SNHL in diabetic patients, our study adds insights into the combined effect of DM and HTN on hearing loss and details the degrees of hearing loss. Differences in methodologies and participant selection, including the inclusion of hypertensive patients in our study, may account for the variations in findings.

Another study by Rolim et al. compared the initial audiometry (A1) in 100 elderly patients with DM and subarachnoid hemorrhage (SAH) followed by subsequent audiometry (A2) following a three to four-year interval to assess the auditory threshold and to ascertain if the rate of hearing loss in these cohorts exceeds that of controls lacking these clinical conditions [12]. The participants were categorized into four gender and age-matched groups: 20 individuals in the DM group, 20 individuals in the SAH group, 20 individuals in the DM/SAH, and 40 individuals assigned to the control group. When comparing the mean auditory thresholds in the initial A1 assessment to the subsequent A2 assessment across the groups, considering the mean increase in auditory thresholds per year, no statistical difference was observed. There was a notable disparity in any frequency between the DM group and its control counterpart; for the SAH group, significant differences were observed at 4 kHz (p = 0.016), 6 kHz (p = 0.013), and 8 kHz (p = 0.037) compared to its control group, as well as a non-significant difference at 3 kHz (p = 0.060); for both DM and HTN groups, significant differences were observed at the frequencies of 500 Hz (p = 0.017), 2 kHz (p= 0.021) and 3 kHz (p < 0.001) between the study group and its control, as well as non-significant differences at 4 kHz (p = 0.058) and 6 kHz (p = 0.066) [12].

Several mechanisms have been proposed to explain the association between DM, HTN, and SNHL, including microvascular and macrovascular complications, oxidative stress, inflammation, and neuropathy [13]. Both DM and HTN cause damage to small blood vessels, leading to microangiopathy in the cochlea, which results in decreased perfusion and hypoxia to the hair cells, stria vascularis, and other cochlear structures [14]. Chronic hyperglycemia and high blood pressure cause endothelial dysfunction, impairing blood flow regulation and increasing vascular permeability. Additionally, DM and HTN contribute to atherosclerosis in larger vessels, which can reduce blood flow to the cochlear arteries and lead to ischemia and oxidative stress. Oxidative damage is caused by hyperglycemia and high blood pressure due to reactive oxygen species (ROS) production in cellular structures of the cochlea. Mitochondria in cochlear cells can become dysfunctional due to excessive ROS, impairing energy production and increasing oxidative stress.

Chronic diseases like DM and HTN often deplete endogenous antioxidants, making the cochlea more susceptible to oxidative damage. These conditions are linked with low-grade inflammation, with elevated pro-inflammatory cytokines such as tumor necrosis factor-alpha (TNF-α), interleukin (IL)-6, and C-reactive protein (CRP) infiltrating the cochlea and promoting inflammatory responses that damage cochlear cells [14-15]. These cytokines can disrupt the blood-labyrinth barrier, leading to an influx of immune cells and further cochlear damage [15].

Chronic hyperglycemia can lead to diabetic neuropathy, affecting the auditory nerve and resulting in impaired signal transmission from the cochlea to the brain [15]. Similarly, HTN can cause hypertensive neuropathy through ischemic injury or direct pressure effects on neural structures, resulting in neuropathy and hearing impairment [14-16]. The combined effects of vascular insufficiency, atherosclerosis, and endothelial dysfunction reduce the efficiency of blood delivery to the cochlea, leading to ischemia and hypoxia [16]. Reduced oxygen delivery to cochlear tissues induces hypoxia, which is detrimental to the survival and function of hair cells and supporting structures in the cochlea [16]. Hypoxia exacerbates oxidative stress and inflammation, creating a vicious cycle of damage [16,17].

Grasping these mechanisms is essential for forging strategies to prevent and manage SNHL in patients with DM and HTN. Early detection of auditory deficits and aggressive management of blood glucose and blood pressure levels may decrease the hazard of hearing loss. Additionally, antioxidant therapy, anti-inflammatory treatments, and measures to improve vascular health could protect against cochlear damage. Highlighting these connections emphasizes the importance of multidisciplinary care in managing these chronic conditions and their complications, ultimately aiming to improve patient outcomes and quality of life. There is a need for increased awareness and education about the risks of SNHL in individuals with DM and HTN. Public health initiatives should inform patients about the importance of maintaining optimal control over their systemic conditions to prevent or delay hearing loss.

Our study has several implications for clinical practice and public health. First, it suggests that diabetic and hypertensive patients should undergo regular hearing screening and evaluation, as SNHL may affect their quality of life, communication, and cognitive function. Second, it indicates that early diagnosis and optimal management of DM and HTN may prevent or delay the onset and progression of SNHL and thus improve these patients' hearing outcomes and prognosis. Third, it highlights the need for more awareness and education about the risk factors and prevention strategies for SNHL among diabetic and hypertensive patients and the general public.

Limitations and strengths of the study

Our study has some limitations that should be considered when interpreting and applying our results. First, we used a cross-sectional design, which does not allow us to establish a causal relationship between DM, HTN, and SNHL or to assess the temporal changes in hearing thresholds over time. A longitudinal or prospective study would be more appropriate to address these issues. Second, we measured only the PTA thresholds, which may not reflect the full spectrum of hearing impairment such as speech discrimination, speech-in-noise, and central auditory processing. A more comprehensive audiological assessment would be more informative and accurate. Third, we did not control for some potential confounding factors such as age, gender, smoking, noise exposure, ototoxic drugs, and other comorbidities that may affect the hearing status of the participants. A multivariate analysis would be more robust and reliable for adjusting these factors.

Despite these limitations, our study has some strengths that enhance its validity and relevance. First, we used a representative sample of diabetic and hypertensive patients and healthy controls with a similar age and gender distribution. Second, we used a standardized and objective method to measure the hearing thresholds, following the recommended guidelines and procedures. Third, we performed a detailed and rigorous statistical analysis, using appropriate tests and parameters to compare the groups and evaluate the correlations.

## Conclusions

Our investigation highlights a notable prevalence of SNHL in individuals with DM and HTN compared to healthy controls. This supports the hypothesis that these conditions may contribute to cochlear damage through mechanisms like microvascular complications, oxidative stress, inflammation, and neuropathy. The severity of SNHL positively correlates with the duration of DM or HTN, highlighting the essence of established disease management on auditory health. Prolonged exposure to the metabolic and vascular changes associated with DM and HTN exacerbates the risk of hearing loss, emphasizing the need for early detection and intervention. Given the crucial role of hearing in communication and quality of life, our study advocates for regular auditory evaluations for patients with DM and HTN. Integrating hearing assessments into routine care can ensure timely identification and management of SNHL, potentially mitigating its progression and enhancing overall well-being.

While our study provides strong evidence linking DM and HTN with SNHL, it acknowledges limitations inherent to its cross-sectional design. Further prospective studies are required to develop causal relationships and observe the progression of auditory decline. A more comprehensive audiological assessment beyond PTA would offer a fuller understanding of auditory deficits in this population. Interdisciplinary approaches to patient care are needed, encompassing metabolic and sensory health, to improve the quality of life for those affected by DM and HTN.
